# Development of Smart pH-Sensitive Collagen-Hydroxyethylcellulose Films with Naproxen for Burn Wound Healing

**DOI:** 10.3390/ph18050689

**Published:** 2025-05-07

**Authors:** Elena-Emilia Tudoroiu, Mădălina Georgiana Albu Kaya, Cristina Elena Dinu-Pîrvu, Lăcrămioara Popa, Valentina Anuța, Mădălina Ignat, Emilia Visileanu, Durmuș Alpaslan Kaya, Răzvan Mihai Prisada, Mihaela Violeta Ghica

**Affiliations:** 1 Department of Physical and Colloidal Chemistry, Faculty of Pharmacy, “Carol Davila” University of Medicine and Pharmacy, 6 Traian Vuia Str., 020956 Bucharest, Romania; elena-emilia.tudoroiu@drd.umfcd.ro (E.-E.T.); lacramioara.popa@umfcd.ro (L.P.); valentina.anuta@umfcd.ro (V.A.); razvan.prisada@umfcd.ro (R.M.P.); mihaela.ghica@umfcd.ro (M.V.G.); 2 Innovative Therapeutic Structures Research and Development Center (InnoTher), “Carol Davila” University of Medicine and Pharmacy, 6 Traian Vuia Str., 020956 Bucharest, Romania; 3 Department of Collagen, Division of Leather and Footwear Research Institute, National Research and Development Institute for Textiles and Leather, 93 Ion Minulescu Str., 031215 Bucharest, Romania; 4 Department of Testing and Quality Control, Division of Leather and Footwear Research Institute, National Research and Development Institute for Textiles and Leather, 93 Ion Minulescu Str., 031215 Bucharest, Romania; madalina.fleancu@yahoo.com; 5 National Research and Development Institute for Textiles and Leather, 16 Lucrețiu Pătrășcanu Str., 030508 Bucharest, Romania; e.visileanu@incdtp.ro; 6 Department of Field Crops, Faculty of Agriculture, Hatay Mustafa Kemal University, 31034 Antakya-Hatay, Turkey; dak1976@msn.com

**Keywords:** smart polymeric dressing, pH-sensitive films, phenol red indicator, colorimetric pH detection, drug release, burn wound healing

## Abstract

**Background**: Developing versatile dressings that offer wound protection, maintain a moist environment, and facilitate healing represents an important therapeutic approach for burn patients. **Objectives**: This study presents the development of new smart pH-sensitive collagen-hydroxyethylcellulose films, incorporating naproxen and phenol red, designed to provide controlled drug release while enabling real-time pH monitoring for burn care. **Methods**: Biopolymeric films were prepared by the solvent-casting method using ethanol and glycerol as plasticizers. **Results**: Orange-colored films were thin, flexible, and easily peelable, with uniform, smooth, and nonporous morphology. Tensile strength varied from 0.61 N/mm^2^ to 3.33 N/mm^2^, indicating improved mechanical properties with increasing collagen content, while wetting analysis indicated a hydrophilic surface with contact angle values between 17.61° and 75.51°. Maximum swelling occurred at pH 7.4, ranging from 5.65 g/g to 9.20 g/g and pH 8.5, with values from 4.74 g/g to 7.92 g/g, suggesting effective exudate absorption. In vitro degradation proved structural stability maintenance for at least one day, with more than 40% weight loss. Films presented a biphasic naproxen release profile with more than 75% of the drug released after 24 h, properly managing inflammation and pain on the first-day post-burn. The pH variation mimicking the stages of the healing process demonstrated the color transition from yellow (pH 5.5) to orange (pH 7.4) and finally to bright fuchsia (pH 8.5), enabling easy visual evaluation of the wound environment. **Conclusions**: New multifunctional films combine diagnostic and therapeutic functions, providing a promising platform for monitoring wound healing, making them suitable for real-time wound assessment.

## 1. Introduction

Wound healing is a highly intricate regenerative process that relies on the coordinated actions of various tissues and cell types [1]. The pH level within the wound environment plays a crucial role in therapeutic wound care, as it not only indicates but also affects key physiological and biochemical processes involved in tissue remodeling [2]. Under normal conditions, the skin’s surface maintains an acidic pH ranging from 4 to 6, which contributes to its protective barrier function and helps prevent microbial colonization [3]. However, this acidic environment can be easily disrupted by wounds, as they lead to the mixing of external skin fluids with the body’s internal fluids, which have a pH of 7.4 [4].

Clinical studies have shown that initially, burns typically exhibit a more alkaline pH compared to unburned skin, with an average pH value between 8.1 and 8.5, due to cellular impairment, exudate accumulation, and bacterial colonization. This alkaline phase promotes bacterial growth and persistent inflammation, which can delay the healing process and increase the risk of infection [5,6,7]. Burn wounds that progress successfully toward healing shift back to a neutral pH and finally to an acidic environment. This acidic medium during the later stages of healing is associated with keratinocyte migration, fibroblast activity, angiogenesis, and epithelialization. Furthermore, an acidic pH promotes the release of oxygen, encourages the enzymatic activity necessary for skin tissue remodeling, and impedes bacterial proliferation [8,9]. These affirmations suggest that pH can serve as a reliable indicator of wound healing [10]. Given its significance, real-time monitoring of wound surface pH could play a crucial role in guiding clinical management and optimizing treatment strategies for improved healing outcomes [11].

Recent studies have explored colorimetric pH sensors for wound monitoring using various pH indicators. Kiti et al. [12] and Petkowska et al. [13] developed colorimetric pH-sensitive wound dressings based on biopolymers and pH indicators (anthocyanins from *Clitoria ternatea* flower and grape, respectively). However, while their systems successfully achieved visual wound pH monitoring, they did not incorporate active drug delivery functionalities. Additionally, neither study comprehensively addressed critical parameters for a wound dressing, such as wetting and swelling capacity, mechanical properties, enzymatic degradation behavior, or drug release kinetics. Liu et al. [14] described a pH-sensitive alginate/polyacrylamide hydrogel wound dressing, loaded with phenol red, achieving rapid color changes, yet lacking wetting behavior, degradation study, and drug delivery functionality to promote wound healing. In contrast, we developed a collagen-hydroxyethylcellulose-based biocomposite with dual function: (1) diagnostic—real-time pH wound monitoring through color change using phenol red and (2) therapeutic—localized controlled release of naproxen (NPX) to actively reduce inflammation and pain, providing a multifunctional platform for burn wound care. We performed a detailed characterization, including swelling tests at different pH levels, enzymatic degradation studies, and drug release profiles, offering a comprehensive physicochemical and biopharmaceutical evaluation not always present in the previously mentioned studies. Finally, we specifically targeted the clinically relevant pH range (5.5–8.5) for burn injuries, linking the dressing color changes with actual healing stages, thereby enhancing the clinical applicability of our system.

This study belongs to a complex research. Collagen-based materials have been widely acknowledged for their outstanding biocompatibility, biodegradability, and ability to promote wound healing [15,16]. As a key component of the extracellular matrix (ECM), collagen plays an essential role in tissue repair and regeneration [17,18]. However, despite its beneficial properties, collagen alone often lacks the necessary mechanical strength and stability for long-term wound coverage. To address these limitations, combining collagen with other polymers can improve its properties [19]. Considering all of the above, in the first stage of our research, we presented an extensive literature screening of cellulose derivative applications as wound dressings addressed for many types of wounds [20]. Starting with the major findings from this previous study, we selected two cellulose derivatives, methylcellulose (MC) and hydroxyethylcellulose (HEC), to combine them with collagen.

The development of polymer-based wound dressings is being studied continuously. The polymeric support has a fundamental role because it directly comes into contact with the wound exudates, which it can properly absorb to maintain a suitable moist environment to promote the healing process [21]. In the last years, multiple polymeric dressings have been developed based on various polymer combinations with clinical applications: human-like collagen, hyaluronic acid, and carboxylated chitosan hydrogels designed as burn wound dressing [22], collagen and gellan gum hydrogels for full-thickness burn injury therapy [23], chitosan nanocomposites for chronic tissue repair [24], hyaluronic acid hydrogels loaded with silver nanoparticles designed to prevent infection, facilitating burn healing [25], and collagen with basic fibroblast growth factor hydrogel, designed as a delivery system for second-degree burn treatment [26].

Considering all the literature research, in the second stage of our research, we designed some spongious matrices by mixing the collagen gel with MC/HEC gels in different ratios. The results showed that collagen and both cellulose derivatives were biocompatible and preserved the collagen native triple helix structure for a maximum content of 30% MC/HEC gels. Moreover, newly designed scaffolds exhibited suitable swelling and wettability capacity to absorb the wound exudates; these findings make these systems optimal supports for drug delivery and wound dressing applications [27].

In the third stage of our extensive research, based on the underexplored potential of HEC and our previous studies’ results, we designed and optimized some spongious drug delivery systems, loaded with naproxen, using a 3-factor, 3-level Box-Behnken design, coupled with response surface methodology. Naproxen, a non-selective non-steroidal anti-inflammatory drug (NSAID), was selected as an anti-inflammatory drug model due to its well-known pharmacological action in managing inflammation and pain, both symptoms being critical in the acute phase (24–48 h) of the healing process of a burn injury [28]. Commonly, NSAIDs, including NPX and their selective cyclooxygenase-2 (COX-2) inhibitors, are described as active substances that inhibit prostaglandin production, reduce inflammation, and act as efficient painkillers [29,30]. Compared to COX-2 selective inhibitors, NPX is more frequently used in topical and localized delivery systems as a result of its suitable permeability and cutaneous tissue retention [31]. Previous studies have strongly formulated this anti-inflammatory drug in hydrogels [32,33,34,35,36], films [37,38], and nanofibers [39] for localized therapy. Accordingly, when NPX is applied topically, it has low toxicity, with limited systemic absorption, which means reduced side effects on the gastrointestinal and cardiovascular levels [32,33]. The use of selective COX-2 inhibitors in topical wound care is limited. Furthermore, regarding the beneficial therapeutic effect on wound healing of selective COX-2 inhibitors, there are few reported studies where some of these drugs (celecoxib and meloxicam) had severe side effects (hepatic toxicity, exaggerated inflammatory processes) on wounded mice [40], a negative impact on wound healing (delaying the reepithelialization and the wound closure) [30,41]. Based on these findings, NPX was incorporated into these collagen-HEC spongious delivery systems to have a localized drug release that can provide targeted therapeutic action while minimizing systemic side effects [42]. That study continued with the optimization process using the Taguchi technique, and four optimal formulations were selected. Their regenerating effect on burn healing was in vivo demonstrated by applying them to experimentally induced burns on Wistar rats (manuscript under review).

Based on the previous results regarding the beneficial anti-inflammatory and epithelializing effects on animals of the optimal formulations, in this study, we developed some smart pH-sensitive films according to the optimal compositions to monitor the pH variation, which can provide important information for burn status and care. The pH indicator dye was phenol red, whose changes in color vary with pH: in acidic pH, it is yellow, in neutral pH it is orange, and in alkaline pH, it is bright fuchsia [43,44]. These novel smart dressings focus on both monitoring burn healing with color transition depending on the pH wound and controlling the inflammation by delivering the anti-inflammatory drug in a controlled manner.

## 2. Results and Discussion

### 2.1. Physical Evaluation of pH-Sensitive Films

The resulting pH-sensitive film dressings were found to be orange-colored because the final pH of the collagen-HEC hydrogels before drying was between 7.2 and 7.4, the physiological pH, where the color of the phenol red indicator is orange. Films were flexible, odorless, without air bubbles, being easily peelable from Petri dishes. One important property of biopolymeric films is their transparency, which is essential for an optimal wound dressing because it allows the physician to monitor the evolution of the healing process [45]. The main physical characteristics of film dressings are presented in Table 1.

The pH-sensitive films developed for burn wound healing and tissue regeneration should have a uniform thickness to ensure consistent mechanical properties [45]. The thickness of the prepared films is primarily influenced by an appropriate homogenization of polymer gels and plasticizers and by a homogeneous distribution of the drug in the film structure. Moreover, the thickness is also controlled by the quantity of hydrogels that are poured into the Petri dishes and by the flatness of the used molds. To ensure a regular distribution of all components, the prepared gels were vigorously mixed before pouring them into the Petri dishes. For every sample, an equal amount of hydrogels was used to cast the pH-sensitive films, and the hydrogels were dried on a flat surface. The results are presented in Table 2. Film thickness values were quite close, ranging from 0.12 mm for F3 to 0.19 mm for F1.

### 2.2. Mechanical Characterization

Mechanical properties of film dressings play a substantial role in establishing the film’s structural integrity during its application at the lesion site, which is a predominant factor in regulating the target wound [46,47]. Film dressings must be able to endure the applied stress on the injury area and must present large resistance to swelling or motion-caused deformation to avoid damage by exudate absorption and by the movement of the patient. The ductility behavior was quantified through the determination of tensile strength [14].

The results obtained from the mechanical testing are shown in Table 2. The values of tensile strength ranged from 0.61 for F1 to 3.33 for F2. The F2 sample with maximum collagen gel and crosslinking agent concentrations presented the highest tensile strength value. The absence of HEC gel and the presence of 100% collagen gel provided a dense and structurally strong membrane, which significantly improved the mechanical strength. This behavior can be assigned to the unique structural characteristics of collagen. Its specific triple-helix configuration, stabilized by extensive hydrogen bonding and covalent crosslinks, contributed to higher tensile strength and structural rigidity [48,49,50]. Moreover, the crosslinking reaction of collagen with glutaraldehyde (GA) strengthened the polymer network by inserting more covalent bonds, which improved the mechanical durability [51]. These molecular interactions demonstrated the higher tensile strength of films based on 100% collagen gel compared to the samples that also contain HEC gel. Glutaraldehyde was used in very low concentrations (0.005 and 0.010%), which completely reacted with the amine groups of collagen, with the formation of new covalent crosslinks. With the entire consumption of glutaraldehyde during the crosslinking reaction, we did not have an excess of the crosslinking agent in the final formulations, so no residuals were predicted to remain. Accordingly, the quantification of glutaraldehyde residuals was not performed. This approach encourages the safety of the newly designed pH-sensitive films for burn wound application, with the maintenance of their structural integrity when they come in contact with the injury exudates.

For F1, F3, and F4 formulations, the mechanical properties are lower than those of F2, this behavior is due to the presence of 15–30% HEC gel. This polymer is more soluble in water and mechanically weaker than collagen, thus reducing the mechanical properties of films compared to F2. Although F1 and F3 had the same polymer composition, F3 had a tensile strength 2.55 times higher than F1. It would be expected that the presence of GA in F1 could increase the tensile strength compared to F3. However, F3 was more resistant to breakage, suggesting that the low GA (0.005%) from F1 was not sufficient to crosslink the collagen molecule in the presence of a high quantity of HEC gel. This phenomenon could be attributed to the possible physical and chemical interactions of HEC with GA crosslinking sites, as presented in previous studies [52,53,54]. Even though F4 had more collagen gel than F1 and the same GA concentration, the tensile strength value of F4 was similar to F1, indicating that the decrease in HEC gel content did not improve the tensile strength.

Taking into account all the findings, it can be concluded that F2 was the strongest film, with the best mechanical properties, being ideal for wound applications where structural stability and mechanical durability are desired. F3 exhibited moderate mechanical traits, with higher elasticity, representing an adequate alternative for flexible wound dressings if some stretchability is required. The least mechanically strong were F1 and F4, but they still can be an option for highly flexible wound dressings, where the structural support is not as necessary.

### 2.3. Scanning Electron Microscopy (SEM)

**Figure 1:**
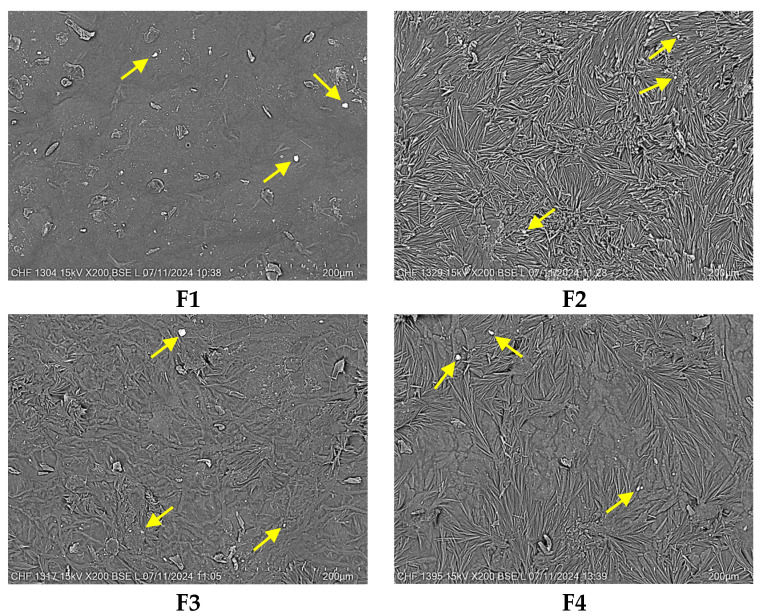
SEM micrographs of pH-sensitive film dressings at 200× magnification, scale bar of 200 μm. Yellow arrows indicated the presence of the drug particles on the surface of the film.

The surface morphology represents a fundamental feature of wound dressings. Accordingly, the surface morphology and the microstructure of the biopolymeric films were examined by scanning electron microscopy. SEM analysis produced high-resolution images that revealed intricate details about the fiber and drug distribution, porosity, and surface topography [55]. This analysis also offered information about the distribution of the drug through the polymeric network, which is important for the drug release performance in the healing process. The micrographs of the designed pH-sensitive film dressings are shown in Figure 1.

Commonly, the morphology of the film dressings should seem homogeneous and regular to establish uniform distribution of the drug into the biopolymeric blend (Figure 1). All formulations appeared to have a quite smooth, compact, nonporous, and continuous surface, suggesting a regular molecular network. The micrographs of the blended films illustrated a homogeneous morphology without cracks, voids, friable areas, agglomerations, or phase separation between the components. The smooth appearance of the films could be attributed to the use of an oven without a vacuum for the samples’ drying [56].

However, small parts of the surface of the films seemed to be slightly rough because, during the drying process, intermolecular and convective forces occurred [57]. This aspect was more obvious when the drug was added, with the presence of its particles on the surface (white spots), which were highlighted through the yellow arrows in the SEM images (Figure 1). The roughness of films could also be influenced by the polymeric composition. Thus, the F2 sample with 100% collagen gel was slightly rougher than the other samples containing 15–30% HEC gel. The appearance of surface roughness could also be assigned to the interaction between the collagen and GA for F1, F2, and F4. GA crosslinking happens principally through the reaction with the free amine groups present in the amino acid residues of collagen [58]. This interaction can lead to microstructural rearrangements and heterogeneity in the film network due to localized differences in crosslinking density, resulting in slight surface roughness [52]. The rough surface of film dressings can have a helpful role in the healing process because it promotes the differentiation, migration, and adhesion of the cells [59]. The rough surface promotes cell adhesion through the formation of more binding sites that enhance the attachment of keratinocytes, fibroblasts, and other cells involved in the restoration process of the skin. Film surface roughness also creates topographical keys that manage cell migration, promoting rapid coverage of the lesion area. Moreover, the rough surface can activate the differentiation of fibroblasts into myofibroblasts, accelerating the injury contraction and consequently its healing [60,61,62].

These findings suggested a high miscibility and mixture homogeneity between collagen and HEC gels, plasticizers, and NPX. Thus, pH-sensitive films seem to fulfill the content uniformity criteria.

### 2.4. Wettability Analysis

**Figure 2:**
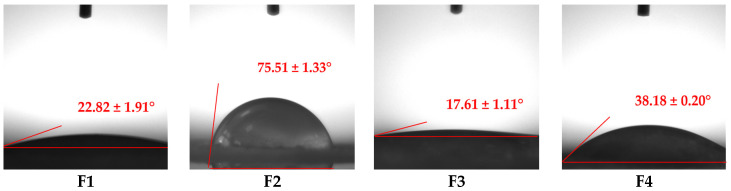
Water droplet contact angles registered on the surface of pH-sensitive film dressings. Values are mean ± SD (*n* = 3).

Wettability is a crucial factor that influences the interaction of wound dressings with biological tissues and the drug release profile [63]. For an ideal dressing, hydrophilic behavior is essential, facilitating the absorption of injury exudates and stimulating the healing process [64]. To investigate the wettability (hydrophilicity or hydrophobicity) of pH-sensitive films, contact angle (CA) is proposed as a valuable evaluation index [65]. When its value is less than 90°, a material has a hydrophilic nature, while a material is considered hydrophobic when the CA value is higher than 90° [51]. Moreover, very low (less than 50°) or very high (higher than 150°) CA values can characterize a material as superhydrophilic or superhydrophobic, respectively [66]. The wetting behavior of pH-sensitive film dressings is illustrated in Figure 2.

According to Figure 2, all films exhibited CA values lower than 90°, confirming their hydrophilic nature. CA values varied from 17.61 ± 1.11° for F3 to 75.51 ± 1.33° for F2.

The combination of two different polymers, collagen (natural) and HEC (semisynthetic), is advantageous because it increases the hydrophilicity of the developed films, as can be seen from the CA values obtained. This behavior can be explained through the following specific features: collagen is a highly hydrophilic polymer due to the presence of multiple polar amino acids in its molecule, which allow it to rapidly swell and retain water, maintaining its structural stability, without dissolving [67]. On the other hand, HEC, a cellulose derivative, also has a hydrophilic nature due to the many hydroxyethyl groups in its molecule, but compared to collagen, HEC is highly water soluble [68]. These findings are also well correlated with the swelling behavior regarding the high swelling capacity and the preservation of the structural integrity of the film dressings.

Considering the previously mentioned, the highest CA was registered for F2 due to the presence of maximum collagen gel and GA concentration, without HEC, and the lowest CA was noticed for F3, the formulation with the maximum content of HEC and without crosslinking agent. The cross-linking of F2 formed a more rigid and durable structure due to the formation of crosslinks between the collagen fibers. This chemical reaction explained the highest CA from all formulations.

The formulations with the maximum quantity of HEC gel (30%), F1 and F3, showed the smallest CA values, having a superhydrophilic nature. F1 had a slightly higher CA than F3 because F1 was crosslinked with GA, enhancing the mechanical strength [69]. The influence of polymer concentration is easily visible when comparing F1 with F4, which had the same concentration of NPX and GA but different concentrations of HEC gel; thus, F4 had a higher CA due to the smaller content of HEC gel (15%) compared to F1 (30%). Even if F4 had a higher CA than F1, it also exhibited superhydrophilic behavior.

Wettability analysis illustrated that the designed film dressings had high hydrophilicity, which is a desired property for the novel designed formulations.

### 2.5. Swelling Behavior

**Figure 3:**
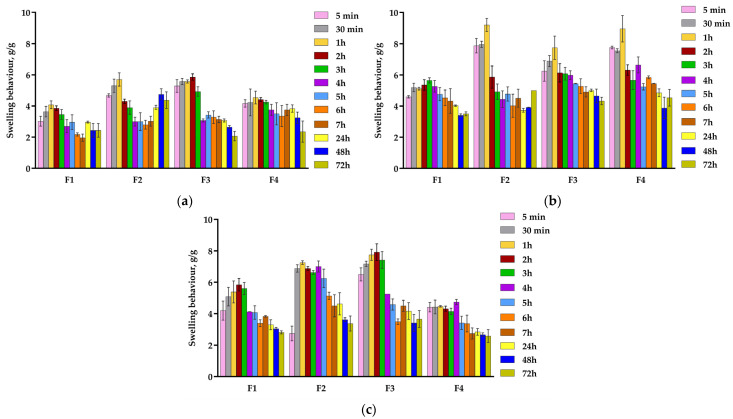
Swelling behavior of pH-sensitive film dressings in different pH media at room temperature for 72 h: (**a**) 5.5; (**b**) 7.4; (**c**) 8.5. Values are mean ± SD (*n* = 3).

Swelling behavior represents a significant aspect of evaluating modern wound dressings because it indicates their capacity to interact with lesions’ exudates under the influence of some external stimuli (pH, temperature, and ionic strength) [59]. An optimal swelling profile ensures the maintenance of a moist environment, prevents dehydration and inflammation, and facilitates painless dressing removal without destroying the newly formed epithelial tissue [70]. Furthermore, swelling behavior directly influences drug release rates and the structural integrity of the films in biological environments [21]. The swelling behavior in three different pH media—acidic for healthy skin (pH 5.5), neutral for the early-stage wound (pH 7.4), and alkaline for the post-burn wound (pH 8.5) [7]—was conducted to observe the pH response of films. The swelling behavior of pH-sensitive films performed for 72 h is shown in Figure 3.

As can be seen in Figure 3, all formulations remained intact even after 72 h in all three phosphate buffer solutions, meaning that the frequency of their removal at the lesion site can be reduced, thus enhancing the patients’ life quality. When a film membrane is immersed in various pH media, it absorbs the solutions and swells. This process is representative of the healing process because wound dressing with an optimal swelling behavior will absorb the lesion exudate when it is applied to the wounded skin. This mechanism facilitates the drug release from the film structure, reducing inflammation and pain, and the collagen-HEC support that remains in the wound area stimulates the healing process [71].

The swelling analysis of all formulations showed distinct behavior due to the various structural and physico-chemical properties, as we explained in the wettability analysis. The combination of collagen with HEC enhanced the hydrophilic nature and the structural integrity of the prepared films [19]. The diffusion process is managed by the attraction between the solvent and the polymer chains [56]. The swelling behavior varied depending on pH, with prominent differences in peak swelling times, with higher swelling at pH 7.4 and 8.5 compared to pH 5.5 due to the differences in the electrostatic interactions between the chemical groups of the polymers and solvent. Generally, all formulations presented the highest swelling at neutral pH, followed by the alkaline and then the acidic ones. These findings confirm their application as wound dressings.

In the neutral medium, carboxylic acid and amide groups from the collagen molecule, respectively, and the hydroxyl groups from the HEC molecule exist in both forms, ionized and non-ionized. Negatively charged carboxylic acid and hydroxyl groups can form hydrogen linkages with both protonated amide groups or molecules of water, increasing the electrostatic repulsion, which causes the highest swelling behavior [72]. According to Figure 3, the highest swelling was recorded for F2 (9.20 g/g), F4 (8.96 g/g), and F3 (7.74 g/g) after 1h, followed by F1 (5.65 g/g) after 3 h.

On the other hand, at pH 8.5, the swelling values were slightly lower than those registered at pH 7.4; this behavior could be due to the deprotonation of functional groups [59]. Thus, the highest swelling was noticed for F3 (7.92 g/g) after 2 h, followed by F2 (7.25 g/g) after 1 h, F1 (5.84 g/g) after 2 h, and F4 (4.74 g/g) after 4 h.

The lowest swelling values were recorded at pH 5.5, where the protonation of the carboxylic acid and hydroxyl groups decreased the swelling behavior because of the stronger intermolecular interactions [59]. All samples showed the maximum swelling after 1 h as follows: F3 (6.07 g/g), F2 (5.70 g/g), F4 (4.54 g/g), and F1 (4.08 g/g).

At each pH level, after these maximum absorption points, each sample registered a consecutive slow decrease, reaching the equilibrium state, which indicated the stabilization of the film structure. These fast maximum absorptions, recorded for the three pH values in a short time interval between one and four hours, are beneficial to managing the burn exudates in the early stages of the wound, thus promoting the healing process.

At all pH values, F1 exhibited lower swelling than F3 (formulations with the same polymer and drug concentration but different GA amounts) due to the crosslinking effect of GA, generating a denser, more compact formulation, impeding the rapid penetration of the solutions through its structure [73]. When F1 is compared with F4 (samples with the same concentration of NPX and GA but different polymer ratios), the influence on swelling behavior of both hydrophilic polymers, but also of the pH media, is noticed. At pH 5.5 and 7.4, from Figure 3, it can be seen that F4 had a slightly higher swelling than F1, but F1 had a higher swelling at pH 8. At every pH, the highest swelling capacity was registered for F3 because it was the only uncrosslinked sample and had the maximum concentration of HEC gel (30%), conferring higher hydrophilicity. Due to the hydroxyl groups from the HEC molecule, the swelling behavior of F3 was the most significant due to the hydrogen bonds that formed with the aqueous solutions of phosphate buffer.

The highest swelling values at pH 7.4 and 8.5 indicate that the obtained formulations could be used as potential dressings for post-burn wounds.

### 2.6. In Vitro Enzymatic Biodegradation

**Figure 4:**
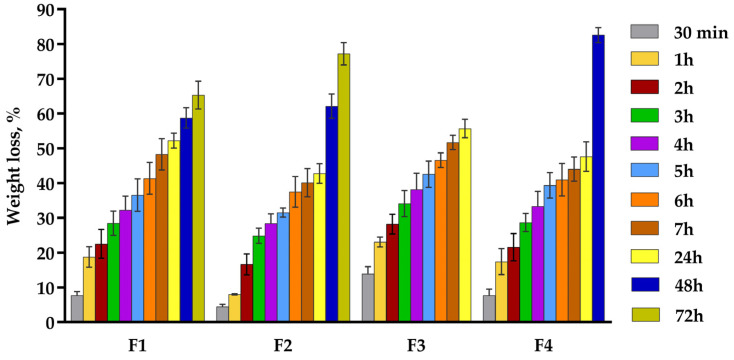
Weight loss of pH-sensitive film dressings in collagenase solution at 37 °C for 72 h. Values are mean ± SD (*n* = 3).

In vitro enzymatic biodegradation is fundamental for evaluating wound dressings, ensuring their degradation rate aligns with the wound healing process [74]. Collagenase, a key enzyme present at the lesion site, degrades the collagen triple helix structure during the inflammatory phase, thereby stimulating tissue remodeling [75]. Moreover, this investigation provides insight into the structural integrity of the pH-sensitive films, a critical factor in determining the appropriate replacement rate of the dressing at the wound site. Figure 4 shows the variation of weight loss (%) over time for polymeric films.

This analysis investigated the residual amount of film dressings after three days of incubation in collagenase solution. According to Figure 4, all samples registered a gradual increase in weight loss, with two of four formulations maintaining their structural stability after three days (F1 and F2), while F3 degraded after 24 h and F4 after 48 h.

The degradation rate of wound dressing has a meaningful effect on the drug release from the polymeric support and also on the replacement frequency of the wounded skin. After 24 h, F1 and F3 degraded more than 50%: 52.23% for F1 and 55.70% for F3. This behavior can be explained by the highest content of HEC gel in their composition, which is a very water-soluble polymer due to the multiple hydroxyl groups that are contained in its structure, being a cellulose derivative. Moreover, higher percentages of disintegration are also due to the small concentration of the crosslinking agent or its absence; thus, the samples are more exposed to degradation by the collagenase. F2 and F4 registered lower weight loss after one day: 42.78% for F2 and 47.64% for F4. The smallest weight loss of the F2 film can be explained by the 100% collagen gel content and the highest amount of GA, which formed a denser structure [73], impeding the accelerated disintegration. F4 had a slightly higher degradation because it had a lower concentration of collagen gel (85%) and of GA (0.005%) compared to F2.

The only uncrosslinked formulation (F2) suffered considerable changes in its structural stability because it presented the highest degradation rate: after 24 h, more than 50% of the total mass was degraded, with a total degradation after 48 h. F2 was followed by F4, which was almost completely digested after 48 h (82.60%), with a total degradation after three days. This comportment can be explained by the small concentration of crosslinking agent (0.005%), but also by the higher amount of collagen. The total disintegration of F3 and the maintenance of structural stability of the other three formulations after 24 h can be explained by the presence of the crosslinking agent, which enhanced the mechanical properties of the films.

### 2.7. In Vitro Drug Release Assay

Inflammation and pain are major challenges during burn healing, causing significant physical and psychological discomfort that negatively impacts the patients’ life quality. Therefore, an appropriate option to reduce these symptoms is NSAIDs like NPX [35].

**Figure 5:**
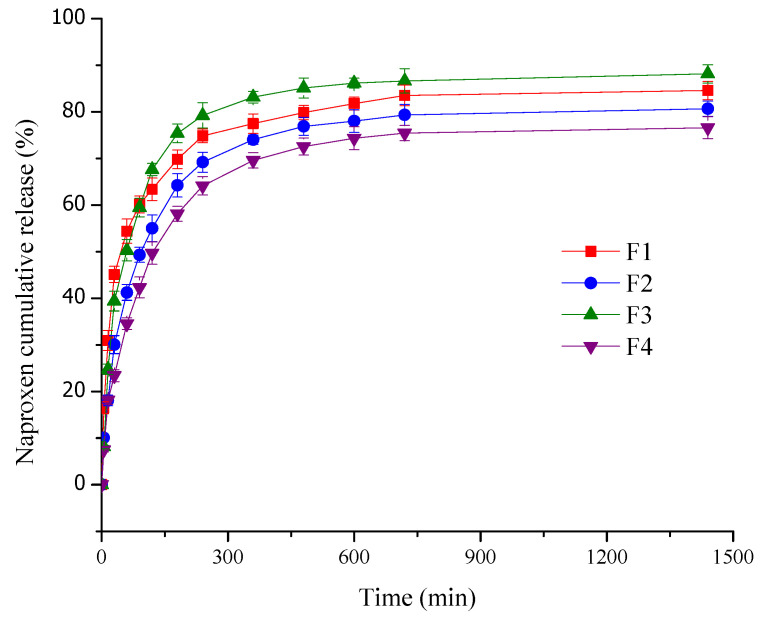
Release profile of NPX from pH-sensitive films for 24 h. Values are mean ± SD (*n* = 3).

The drug release from the pH-sensitive films varied from fast to slow and prolonged trends depending on the film nature and characteristics (polymeric composition, thickness) and also on the dissolution media [76]. The drug release profile is presented in terms of cumulative percentage for 24 h in Figure 5.

The pH-sensitive films exhibited a biphasic drug release profile over 24 h (Figure 5). Initially, a burst release occurred within the first hour due to loosely interacting or weakly bound NPX on the film surface, as confirmed by SEM analysis. This fast release was succeeded by a slower and sustained release phase for the next 24 h [57]. This behavior is explained by the remaining drug fraction incorporated within the polymeric film. Thus, NPX is slightly released when the hydrated polymeric support begins to swell and the drug diffuses through the loose swollen membrane [70]. A complex is formed between the polymeric matrix and drug molecules (“host-guest complex”), leading to a delay in the drug delivery [21].

According to Figure 5, in the first hour, the highest burst release (54.39%) was registered for F1, closely followed by F3 (50.29%). The highest burst release values for both samples were due to the maximum content of HEC gel (30%), a very hydrophilic polymer. A lower level of HEC gel and an increase in collagen gel in the formulations led to a decrease in burst release values for F2 (41.24%) with 100% collagen gel and, respectively, F4 (34.54%) with 15% collagen gel. After the first hour, a slower and sustained drug release was recorded for over 24 h. After this period, the cumulative NPX release percentages varied from 76.60% for F4 to 88.19% for F3. As we explained above, at the burst release effect, the values recorded after 24 h are also strongly influenced by the composition of the film. Thus, the highest values were obtained for F3 and F1 due to the highest concentration of HEC gel (30%). Moreover, F1 had a slower NPX release than F3 because it was crosslinked with GA, resulting in a more compact network that slowly delivers the drug in the medium. F2 and F4 registered lower values than F1 and F3, with the lowest value for F4; this behavior is explained by the reduction in the concentration of HEC gel: 0% for F2 and 15% for F4. An immediate release of NPX is beneficial for quickly reducing the acute symptoms of inflammation and pain that occur immediately after the skin is burned. Moreover, equally significant is the slower and more controlled release of the anti-inflammatory drug in the later hours after the initial injury because the first 24–48 h correspond to the inflammatory period of the burn healing process [77]. Such combined effects substantially promote the patient’s quality of life and induce a reduction of the wound dressing exchange and consequently a reduction of treatment costs [78].

Several studies have explored the NSAIDs release behavior from biopolymeric films intended for wound healing. Thu et al. [79] developed an alginate-bilayer hydrocolloid film, releasing ibuprofen slowly over 8 h due to a swelling-controlled mechanism. Maver et al. [78] reported some cellulose-based films with a fast burst release of diclofenac in the first 5 min, followed by a stationary phase for 4 h. Jantrawut et al. [57] evaluated low-methoxyl pectin-based films with different plasticizers for indomethacin release and found that over 50% of the drug was released in the first 120 min, with no significant differences between formulations regardless of the plasticizer used. Mishra et al. [38] demonstrated that chitosan-based films exhibited a burst release in the first 120 min, with 20–60% NPX released. Moreover, the addition of polyethylene glycol led to a sustained release of NPX for over 24 h. Bernardo et al. [37] achieved a delayed NPX release from alginate-hydroxyapatite-based films for over 48 h. Compared to these systems, our collagen-HEC films offer a biphasic release profile that can properly handle the burn treatment: an initial burst release to control acute inflammation and pain, followed by a prolonged drug delivery for over 24 h, which ensures an optimal anti-inflammatory effect for this period, corresponding to the inflammatory phase of the healing process.

To set up the release mechanism of NPX from the pH-sensitive films, the experimental data were fitted with two kinetic models: Higuchi and Power Law. To evaluate the goodness of fit for every previous kinetic model, two fundamental metrics are used: correlation coefficient (R) and adjusted R squared (R^2^). The last indicator is important when the kinetic models are compared because, besides goodness of fit assessment, adjusted R squared also penalizes the mathematical models for their complexity. This metric is fundamental when kinetic release models are compared regarding the number of predictors: the Power Law model has more parameters (diffusional exponent, n) than Higuchi [80]. Table 3 indicates the values of the correlation coefficient and adjusted R squared.

According to Table 3, the highest R and adjusted R^2^ values were recorded for the Power Law model, which means that the experimental release data were best fitted by this mathematical model. R values ranged between 0.9508 and 0.9675, while the adjusted R^2^ values varied from 0.8915 to 0.9310. Compared to the other kinetic model, the Higuchi, Power Law model had the highest values of both important metrics (R and adjusted R^2^), indicating that this kinetic model is considered the best model to fit the experimental data, considering the Power Law model complexity.

The kinetic model ranking based only on accuracy, including the comparison of correlation coefficient values, is not sufficient because the model variability is not described. Therefore, the kinetic model comparison and evaluation were performed using the Akaike Information Criterion (AIC) metric. This tool also indicates the model variability (precision), in addition to the goodness of fit (accuracy) [81]. For this study, we used the AIC_c_ (small-sample corrected AIC) values, taking into account the small number of experimental points. The kinetic mathematical model that best represents statistically the NPX release is the model that presents the lowest AIC_c_ values [82].

Table 3 shows that the lowest AIC_c_ values were registered for the Power Law model, being lower than the AICc values registered for the Higuchi model. This aspect means that the Power Law model is considered the best model to fit the experimental release data, considering the model’s complexity. Considering all mentioned, the highest values of R and adjusted R^2^ and the smallest values of AIC_c_ were used to choose the kinetic mathematical model that best fit the experimental data. The kinetic model that fulfilled these characteristics was the Power Law model, which properly explained the NPX release mechanism from the pH-sensitive films.

As can be seen in Table 3, the diffusional exponent values varied from 0.22 to 0.28, which indicates a non-Fickian drug transport [83], which includes three various mechanisms: firstly, the NPX release from pH-sensitive film surface; secondly, the drug diffusion from the swollen films; thirdly, the NPX release due to the polymeric scaffold erosion [84].

### 2.8. Colorimetric Analysis

**Figure 6:**
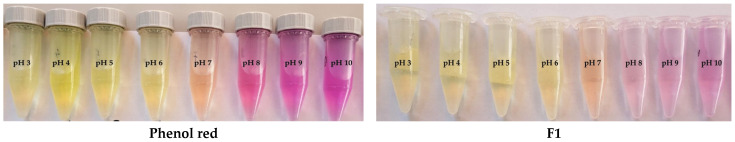

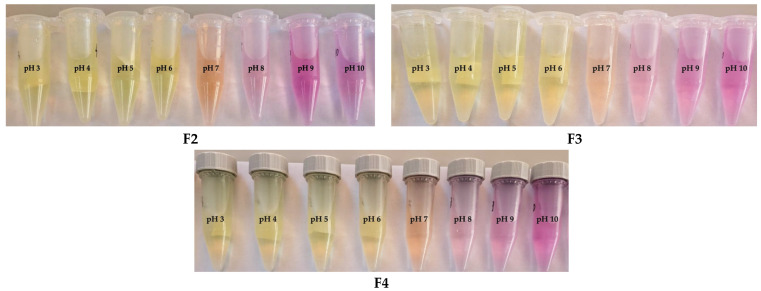
Image of pH-sensitive film dressings in buffer solutions with pH 3 to pH 10.

In the development of pH-sensitive materials for wound pH monitoring, it is essential to ensure that the color change can be distinguished by the naked eye [3]. Collagen-HEC films loaded with phenol red as a pH indicator were analyzed in different pH buffer solutions to simulate the clinical environment [72]. Their color modifications were captured by a camera, as can be seen in Figure 6.

As can be seen in Figure 6, as the pH shifted from 3 to 10, the film dressings transitioned from yellow (pH lower than 6.8) to orange (pH 6.8–7.5) and finally to fuchsia (pH higher than 7.5), clearly visible to the naked eye. The color modifications furnished valuable insights. Thus, the pH interval of the color changes matches the pH window, which is required to indicate the status of a burn wound. As the injury heals, its environment progresses from an alkaline form to a neutral one and later to an acidic status [14]. Table 4 presents the color parameters of the pH-sensitive films in pH 3–10 buffer solutions.

According to Table 4, for all formulations, a* values increased with the increase in pH: from 0.15 at pH 3 to 3.94 at pH 10 for F1, from −0.31 at pH 3 to 3.70 at pH 10 for F2, from −0.32 at pH 3 to 4.44 at pH 10 for F3, and from −0.72 at pH 3 to 4.09 at pH 10 for F4. This aspect indicated that the films’ color gradually turned redder at higher pH, consistent with phenol red behavior. On the other hand, b* became more negative with the increase in pH: for F1 from −8.20 at pH 3 to −14.40 at pH 10, for F2 from −7.93 at pH 3 to −14.19 at pH 10, for F3 from −7.39 at pH 3 to −14.75 at pH 10, and for F4 from −7.12 at pH 3 to −14.05 at pH 10, suggesting that the color progressively turned to blue/purple tones [85]. For F1, ΔE registered a visible increase from 1.60 (pH 3) to 4.41 (pH 4), showing early sensitivity. Then, the ΔE value slowly decreased with the increase in pH but also remained visible to the naked eye. F2 exhibited an increase in ΔE at pH 4–5, followed by a decrease at the next pH levels, while F3 registered a sharp jump from pH 3 to pH 4 and 5, where the ΔE value was about 3.5, meaning the sample is quite responsive in the acidic medium. The same trend was observed for the F4 sample, with a visible increase in ΔE from 1.42 (pH 3) to 3.33 (pH 5). Larger ΔE values signify that the pH-sensitive films can have proper color variation according to the pH value, which suggests adequate visual color variability [86]. The color changes of biopolymeric films were perceptible to the naked eye in multiple pH solutions, indicating that the designed films have a sensitive response to pH modification. Taking into account that the pH-sensitive films will be handled as wound dressings and the average pH of an injury is around 7, the color of the wound dressing will turn orange. When the skin is burned, the local pH will increase to around 8–8.5, and the color of the film dressing will change to fuchsia. If the burn injury heals, the film color will progressively change to yellow because normal skin has a pH value close to 5.5 [87].

These color transitions of the films, when applied at the lesion site, represent an excellent indicator of wound status, guiding the physicians to properly choose the treatment for rapid and effective healing.

## 3. Materials and Methods

### 3.1. Materials

Calf skin was used as the primary source to extract type I fibrillar collagen in gel form using the method that was set up by the Collagen Research Department of the Leather and Footwear Research Institute Division from the National Research & Development Institute for Textiles and Leather, Bucharest, Romania [88]. The initial collagen gel had a 2.43% concentration (*w*/*w*), and the pH was acidic. Hydroxyethylcellulose (analytical grade) was acquired from Sigma-Aldrich, Steinheim, Germany, and had a viscosity of 4000 cPs. Naproxen ((S)-(+)-2-(6-Methoxy-2-naphtyl)propionic acid, 99% purity) was obtained from Thermo Fisher, Kandel, Germany, and type I collagenase (*Clostridium histolyticum*, analytical grade) was purchased from Sigma-Aldrich, St. Louis, MO, USA. Orthophosphoric acid (85% analytical grade), phenol red (analytical grade), glutaraldehyde (25% aqueous solution, ≥98% purity), and sodium hydroxide (analytical grade) were provided by Merck, Darmstadt, Germany. Disodium hydrogen phosphate, potassium dihydrogen phosphate, ethanol absolute, and glycerol (analytical grades) were supplied by Chemical Company S.A., Iași, Romania. The distilled water was used for sample preparation and different analyses.

### 3.2. Methods

#### 3.2.1. Preparation of Biopolymeric Films

The collagen gel used for the preparation of the film dressings had a concentration of 1.1% (*w*/*v*) and a neutral pH (7.2–7.4), being obtained from the initial acid collagen gel of 2.43% concentration with distilled water and 1 M sodium hydroxide under continuous mixing with a mechanical stirrer. To prepare a 3% HEC hydrogel, the HEC powder was dispersed in distilled water at room temperature under continuous stirring until the hydrogel was obtained. Consequently, collagen and HEC hydrogels were mixed in different ratios; then, NPX was added in various concentrations according to Table 5.

To these formulations, a mixture of two plasticizers, ethanol absolute (3 mL) and glycerol (0.3 mL), was added under mechanical stirring to obtain a homogenized mixture. The pH of the samples was adjusted to the physiological one, varying between 7.2 and 7.4 using sodium hydroxide solution (1M). After this important step of pH regulation, a small volume (1.5 mL) of 0.02% phenol red aqueous solution was added, resulting in orange-colored hydrogels (color of the phenol red at neutral pH). The final stage of the preparation process was the crosslinking of three hydrogels with 0.005% or 0.010% glutaraldehyde, one sample remaining uncrosslinked. Hydrogels were then poured into 5 cm Petri dishes and maintained in the oven at 33 °C to obtain the dried films. When completely dry, the films were removed from the Petri dishes very carefully and placed in plastic bags individually for further analysis. The compositions were named F1, F2, F3, and F4. The biopolymeric films obtained were characterized by multiple analyses: physical characterization, mechanical properties, scanning electron microscopy, water contact angle test, swelling behavior, in vitro enzymatic degradation, drug release kinetics, and colorimetric analysis.

#### 3.2.2. Physical Evaluation of pH-Sensitive Films

The designed pH-sensitive film dressings were physically investigated by visual inspection for their color, odor, air bubbles, transparency, flexibility, ease of difficulty regarding their peelability from Petri dishes, and thickness. Transparency was investigated by putting the films over a printed text and evaluating if the text remained readable without compelling blurring. We considered the films to be transparent because the text was readable [45]. To evaluate the presence of air bubbles, we placed them in an ambient light. The films did not present any form of air bubbles. Flexibility was assessed by performing the folding endurance test. Accordingly, this analysis was performed by manually folding every formulation repetitively in the same place until it cracked. Films that can be folded more than 300 times without cracking were presumed to pass the folding test [70]. Our formulations successfully passed this test, indicating proper flexibility to be attached to the wounded skin without cracking. All our formulations were considered peelable because they were easily removed from the Petri dishes without fracturing and adhering to the casting molds [89].

#### 3.2.3. Mechanical Characterization

To evaluate the mechanical properties of pH-sensitive film dressings (film thickness and tensile strength), the samples were analyzed according to an actual standard, SR EN ISO 3376:2020 *Determination of tensile strength and percentage elongation* [90]. Tests were conducted in the Leather and Footwear Research Institute (ICPI) accredited laboratory, using the traction test machine “Tinius Olsen” (India).

#### 3.2.4. Scanning Electron Microscopy (SEM)

The morphology of the film’s surface was analyzed by a Tabletop TM 4000 Plus (Hitachi, Japan) scanning electron microscope using small pieces of dried films, at 15 kV accelerating voltage, in a low vacuum mode, and a backscattered electron detector. Microstructural characteristics of dried formulations were studied without the coverage of the samples with a conductive layer. Micrographs were captured at 200× magnification, with a scale bar of 200 μm.

#### 3.2.5. Wettability Analysis

The water static contact angle of the biopolymeric films was measured at room temperature using a CAM 101 goniometer (KSV Instruments Ltd., Espoo, Finland). Briefly, the film was placed on a glass slide, and the distilled water was cautiously dropped from a Hamilton syringe onto the dried film surface. The moment when the water droplet came into contact with the film surface was captured with a digital video camera. Contact angles were measured three times on different zones of each surface and averaged.

#### 3.2.6. Swelling Behavior

The gravimetric method [91] was used to investigate the swelling behavior of the dried films at different pH levels of phosphate buffer solutions: acidic, neutral, and alkaline (pH 5.5, 7.4, and 8.5). The films were cut into a square shape (0.25 cm^2^), and the initial weight was accurately measured (W_0_). The small pieces were subsequently soaked in 3 mL of pH media at room temperature. Then, swollen film pieces were removed from the different media after specific time intervals (30 min, 1, 2, 3, 4, 5, 6, 7, 24, 48, and 72 h) and weighed again at room temperature on a digital balance (W_s_). The experiment was conducted three times to obtain precise results. The swelling behavior of the dried films was calculated using Equation (1) [72]. For every formulation, the mean and standard deviation were estimated.

(1) $$Swelling\;behaviour\;(g/g)=\frac{W_{s}-W_{0}}{W_{0}}$$

#### 3.2.7. In Vitro Enzymatic Biodegradation

The in vitro enzymatic degradation of films was assessed by measuring weight loss over time in a collagenase solution. Identically shaped film samples with similar initial weight were first immersed in a solution of phosphate buffer (pH 7.4) for 24 h to reach equilibrium. Once fully hydrated, the swollen samples were weighed (W_0_). Subsequently, they were transferred into a 3 mL solution of phosphate buffer (pH 7.4) containing collagenase (10^−6^ μg/mL) and incubated at 37 °C (simulation of physiological conditions) for various time intervals: 30 min, 1, 2, 3, 4, 5, 6, 7, 24, 48, and 72 h. After each incubation period, the film pieces were pulled out and weighed (W_t_). Each measurement was performed in triplicate, and the average value was determined. The percentage weight loss of the polymeric films was then calculated following Equation (2):

(2) $$Weight\;loss\;(\%)=\frac{W_{0}-W_{t}}{W_{0}}\times 100$$

#### 3.2.8. In Vitro Drug Release Assay

The in vitro drug delivery was performed using the paddle dissolution apparatus (Vision G2 Classic 6 Dissolution Tester, Hanson Research, Chatsworth, CA, USA) and the corresponding disks for testing topical membranes (d = 35 mm, mesh 40, Sotax, Orléans, France). NPX-loaded pH-sensitive films with a 5 cm diameter were placed onto the disk. The resulting excess was removed, and the obtained films were weighed and assembled in the disk. The pH of wound fluid is similar to a physiological buffer solution (pH 7.4), considering the ion content and pH value [92]. Accordingly, the release characteristics of NPX from collagen-HEC films were studied in phosphate buffer solution (PBS, pH 7.4, heated at 37 ± 0.5 °C), simulating the physiological conditions of a real wound and plasmatic environment at 50 rpm [51]. When the medium temperature reached 37 °C, the disks were placed into the vessels of the tester. At specific time intervals (5, 15, 30, 60, 90, 120, 180, 240, 360, 480, 600, 1440 min), 5 mL of each sample was taken with a syringe and replaced with 5 mL of fresh PBS (37 °C) to maintain the volume of release medium in the vessels. The standard calibration curve (R^2^ = 0.9996) was used to calculate the NPX concentration in the release medium at an absorbance of λ = 262 nm, using a UV–Vis spectrophotometer (Jasco, Tokyo, Japan). The drug release assay was performed in triplicate for 24 h.

Analyzing the NPX release using adequate mathematical models facilitates a better understanding of the underlying release mechanisms. To identify the mechanism governing the NPX release from the pH-sensitive film dressings, the cumulative release data were fitted with two models: the Power Law model (Equation (3)) and its specific case—Higuchi model (n = 0.5):

(3) $$\frac{M_{t}}{M_{\infty }}={kt}^{n}$$

where: M_t_ and M_∞_ are described as the released amount of NPX at t time and equilibrium, M_t_/M_∞_ is the fractional release of NPX in t time, “k” is the release rate constant characteristic to collagen-HEC-NPX system (expressed in 1/min^n^), and “n” is defined as the diffusional exponent specific to the drug release process (dimensionless).

#### 3.2.9. Colorimetric Analysis

Color changes of pH-sensitive films were analyzed in the pH range of 3 to 10. Films were immersed in the buffer solutions and stirred for 1 min. To determine their colorimetric response, soft cotton materials were submerged in the previously obtained colored solutions. Materials were left for a few minutes in the solutions, then were taken out and left at room temperature to dry. The color of the impregnated cotton materials was investigated by a UltraScan^®^ PRO spectrophotometer (Hunter Associates Laboratory Inc., Reston, VA, USA). To correlate the color changes, CIE (International Commission Illumination) color space coordinates were measured. Mention that L* indicates lightness (+) and darkness (−), a* corresponds to redder (+) and greener (−), and b* suggests yellower (+) and bluer (−) [93]. For each film dressing, the total color difference (ΔE) was calculated according to Equation (4) [44].

(4) $$\Delta E=\sqrt{{\left(\Delta L\right)}^{2}+{\left(\Delta a\right)}^{2}+{\left(\Delta b\right)}^{2}}$$

## 4. Conclusions

This study was focused on the development of some pH-sensitive film dressings based on collagen, hydroxyethylcellulose, and naproxen, loaded with a pH indicator, phenol red, to be used for the pH detection of a wound and to indicate the healing status. The films demonstrated desirable physicochemical and functional properties, including optimal flexibility, peelability, and hydrophilicity to support their use for burn wound application. The films exhibited proper mechanical and swelling properties, with variations dependent on the polymeric composition, supporting their capacity to absorb the exudates that form at the lesion site while maintaining their structural integrity for more than 24 h. The high capacity to absorb lesion exudates favors the drug release from the polymeric membrane and rapid local effect at the beginning of the burn when the inflammation is very high. The naproxen release from the pH-sensitive films showed a biphasic profile: the burst release effect in the first hour with an immediate drug release, followed by a sustained and prolonged release for the next 24 h, ensuring a suitable control of acute inflammation and pain. Moreover, the pH-sensitive color changes from yellow (acidic pH) to fuchsia (alkaline pH) across a pH interval of 3–10 furnished a visually perceptible indicator of environmental pH, a meaningful characteristic for real-time monitoring of injury status.

While these outcomes are promising for burn wound healing, one limitation of this study is the lack of evaluation of the films’ biological safety. Future studies will include in vitro cytotoxic assessment, in vivo validation using murine burn models, and process scalability of newly pH-sensitive films to support their clinical application. Concluding, the dual function of the designed films positions them as strong options with considerable potential for real-time wound control and therapeutic applications.

## Figures and Tables

**Table 1:** Physical evaluation of pH-sensitive film dressings.

Film Code	Transparency	Air Bubbles	Flexibility	Peelability
F1	*✓*	×	*✓*	*✓*
F2	*✓*	×	*✓*	*✓*
F3	*✓*	×	*✓*	*✓*
F4	*✓*	×	*✓*	*✓*

**Table 2:** Thickness and mechanical characterization of pH-sensitive film dressings.

Film Code	Thickness (mm)	Tensile Strength (N/mm^2^)
F1	0.19	0.61
F2	0.16	3.33
F3	0.12	1.56
F4	0.16	0.62

**Table 3:** Kinetic parameters registered for different kinetic mathematical models; cumulative NPX released percentages.

Films Code	Higuchi Model			Power Law Model			Kinetic Constant, k (1/min^n^)	Diffusional Exponent, n	Drug Released (%)
	R	Adj R^2^	AIC_c_	R	Adj R^2^	AIC_c_			
F1	0.8502	0.6999	81.30	0.9675	0.9310	60.73	0.228	0.22	84.59
F2	0.8870	0.7692	78.79	0.9601	0.9154	64.74	0.147	0.26	80.68
F3	0.8527	0.7050	84.82	0.9508	0.8915	70.81	0.196	0.23	88.19
F4	0.9028	0.7998	75.83	0.9616	0.9186	63.23	0.120	0.28	76.60

**Table 4:** Color parameters of pH-sensitive films immersed in different pH buffer solutions.

	F1				F2				F3				F4			
pH	L*	a*	b*	ΔE	L*	a*	b*	ΔE	L*	a*	b*	ΔE	L*	a*	b*	ΔE
3	92.03	0.15	−8.20	1.60	92.52	−0.31	−7.93	2.82	91.92	−0.32	−7.39	0.55	92.47	−0.72	−7.12	1.42
4	92.04	1.91	−11.29	4.41	91.80	1.32	−9.83	3.09	91.63	1.64	−10.45	3.82	92.16	1.13	−9.59	2.95
5	91.82	2.05	−11.14	3.25	91.83	1.71	−10.20	3.13	91.65	1.97	−10.74	3.35	92.42	1.65	−10.35	3.33
6	91.76	2.39	−12.11	2.31	92.34	2.25	−10.90	1.83	91.81	2.37	−11.19	1.55	91.62	1.78	−8.61	1.42
7	91.96	2.64	−12.46	2.18	92.24	2.64	−10.71	0.92	91.84	3.04	−11.66	1.18	91.95	2.29	−9.79	1.19
8	92.60	3.07	−12.89	2.68	91.75	3.26	−13.17	1.97	91.56	3.38	−12.07	2.68	91.57	3.12	−12.67	2.56
9	91.54	3.53	−13.92	2.02	91.56	4.16	−14.18	2.34	91.72	3.96	−13.24	2.90	91.67	3.30	−14.02	2.08
10	91.42	3.94	−14.40	2.41	90.71	3.70	−14.19	1.68	90.73	4.44	−14.75	1.24	91.54	4.09	−14.05	1.54

**Table 5:** Composition of the different dried films.

Film Code	Coll/HEC ^1,2^, (g%/g%)	NPX ^2^ (g%)	GA ^2^ (g%)
F1	70:30	1.00	0.005
F2	100:0	0.50	0.010
F3	70:30	1.00	0.000
F4	85:15	1.00	0.005

^1^ The ratio between collagen and HEC gels. ^2^ Quantities of Coll/HEC, NPX, and GA are reported to 100 g gel.

## Data Availability

The data presented in this research are available in the article.
